# Supplementary material for: Impact of Straw Incorporation on the Physicochemical Profile and Fungal Ecology of Saline–Alkaline Soil
Source: Microorganisms. 2024 Jan 28;12(2):277. doi: 10.3390/microorganisms12020277 (PMC10892582; doi:10.3390/microorganisms12020277)
Supplement: Supplementary file 1 [file microorganisms-12-00277-s001.zip › microorganisms-2793219-supplementary.pdf]

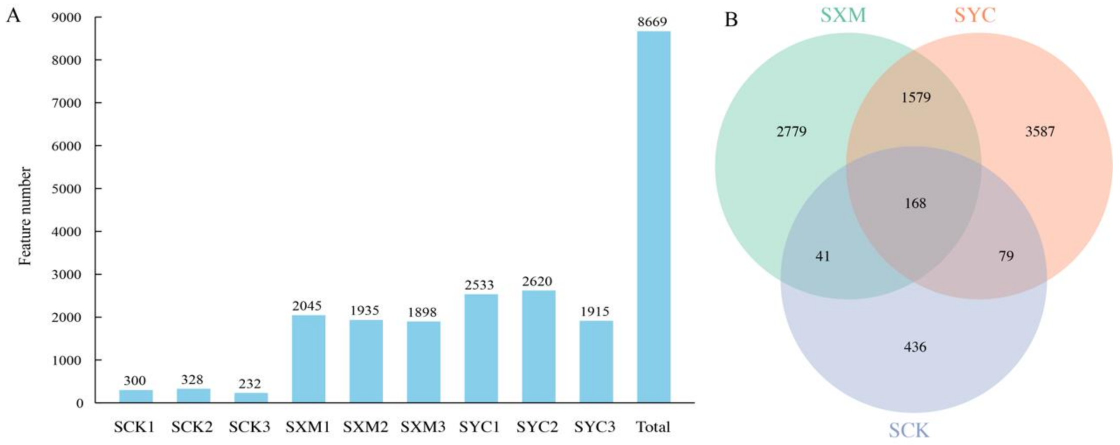

Figure S1: Analysis of OTU number distribution of fungi. (A) the distribution map of characteristic numbers among treatments, in which SCK1, SCK2, and SCK3 represent 0-10cm, 10-20cm, and 20-30cm of three different soil depths in saline-alkali soil control groups, respectively. (B) characteristic Venn diagram.

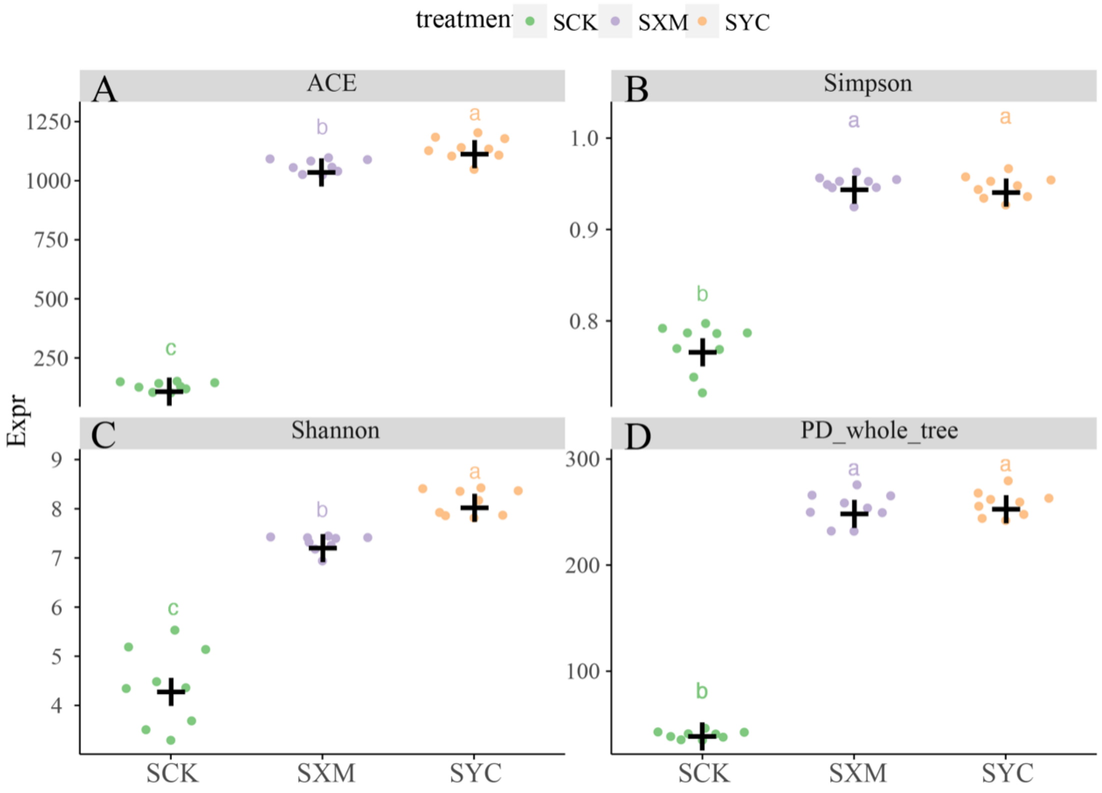

Figure S2: Alpha diversity of soil fungal community under different treatments. (A) Abundance-based Coverage Estimator. (B) Simpson Index. (C) Shannon-Wiener index. (D) The diversity index is calculated by phylogenetic trees. Lowercase letters indicate the significance between treatments ($P \leq 0.05$).

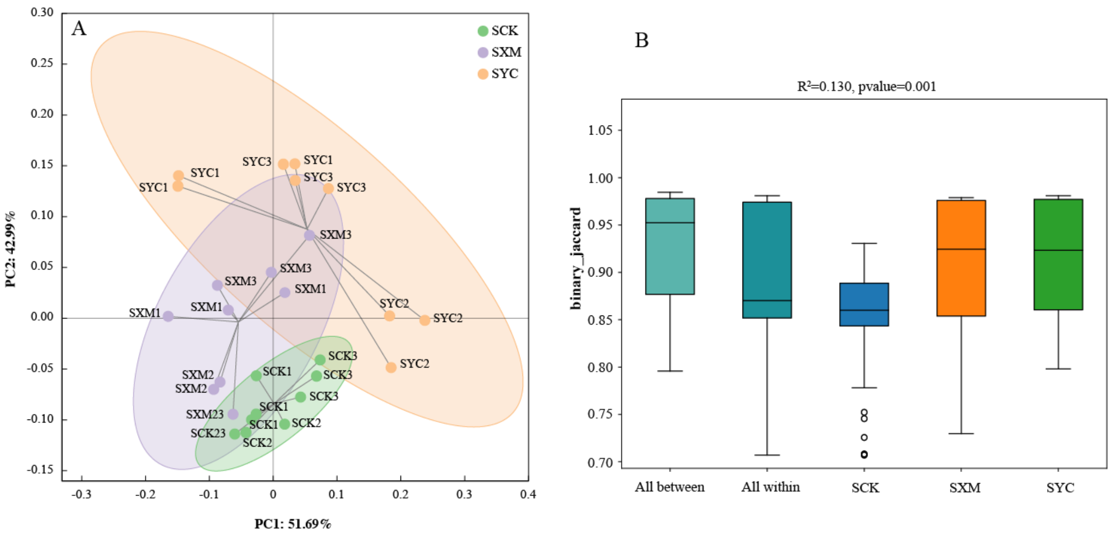

Figure S3: (A) Beta diversity of soil fungal community under different treatments. (B) PERMANOVA analysis box diagram, The box plots above "All between" represent the Beta distance data for all intergroup samples. "All within" upper box plots represent all intra-group sample Beta distance data. The latter box plots represent the Beta distance data between samples within groups for different subgroups, respectively.
